# Retraction Note: Isovitexin reduces carcinogenicity and stemness in hepatic carcinoma stem-like cells by modulating MnSOD and FoxM1

**DOI:** 10.1186/s13046-022-02470-7

**Published:** 2022-08-30

**Authors:** Xiaocheng Cao, Lihua Liu, Qing Yuan, Xiang Li, Yinghong Cui, Kaiqun Ren, Chang Zou, A. Chen, Chang Xu, Yebei Qiu, Meifang Quan, Jiansong Zhang, Jianguo Cao, Xiangding Chen

**Affiliations:** 1 Department of Pharmaceutical Science, Medical College, Hunan Normal University, Changsha, 410013 Hunan China; 2 Key Laboratory of Study and Discover of Small Targeted Molecules of Hunan Province, Changsha, 410013 Hunan China; 3 Laboratory of Molecular and Statistical Genetics, College of Life Sciences, Hunan Normal University, Changsha, 410081 Hunan China; 4 Pharmacy Department, The Second Clinical Medical School of Jinan University, Shenzhen People’s Hospital, Shenzhen, 518020 China; 5 Shenzhen Public Service Platform on Tumor Precision Medicine and Molecular Diagnosis, Shenzhen People’s Hospital, Shenzhen, 518020 China; 6 Department of Preclinical Medicine, Medical College, Hunan Normal University, Changsha, 410013 Hunan China; 7 Clinical Medical Research Center, The Second Clinical Medical School of Jinan University, Shenzhen People’s Hospital, Shenzhen, 518020 China

**Retraction Note: J Exp Clin Cancer Res 38, 264 (2019)**

**https://doi.org/10.1186/s13046-019-1244-6**

The Editor-in-Chief has retracted this article. Concerns were raised regarding a number of figures, specifically:

1. Figure 1b: the panel for MHCC97H appears to overlap with the + panel for AD-GFP in Figure 5f.
2. Figure 1c: the panel for HCSLCs appears to overlap with the + panel for Ad-FoxM1 in Figure 5g.
3. Figure 2b: the panel for Ad-GFP appears to be identical with the right hand panel of Figure 6b.
4. Figure 3I of S1: the panels for CD133/20 and CD133/10 appears to be identical with the panels for /- and -/ for IL-8 in Figure 9D in [1]

The authors were unable to provide uncropped original image for this article. Therefore, the Editor no longer has confidence in the integrity of the data.

Meifang Quan, Jiansong Zhang and Kaiqun Ren agree to this retraction. Xiaocheng Cao, Lihua Liu, Qing Yuan, Xiang Li, Yinghong Cui,Chang Zou, A. Chen, Chang Xu, Yebei Qiu, Jianguo Cao and Xiangding Chen have not responded to any correspondence from the editor about this retraction.
